# Fiber-Optic Skew Ray Sensors

**DOI:** 10.3390/s20092499

**Published:** 2020-04-28

**Authors:** George Y. Chen, Jinyu Wang, David G. Lancaster

**Affiliations:** 1 Laser Physics and Photonic Devices Laboratories, School of Engineering, University of South Australia, Mawson Lakes, 5095 SA, Australia; david.lancaster@unisa.edu.au; 2 Key Laboratory of Optical Fiber Technology of Shandong Province; Qilu University of Technology (Shandong Academy of Sciences), Jinan 250014, China; jinyu.wang@sdlaser.cn

**Keywords:** skew ray, multimode fiber, evanescent field, sensor, ray optics

## Abstract

The evanescent fields along multimode fibers are usually relatively weak. To enhance the sensitivity of the resulting sensors, skew rays have been exploited for their larger number of total internal reflections and their more comprehensive spread over the fiber surface. The uniform distribution of light–matter interactions across the fiber surface facilitates high sensitivity through an increased interaction area, while mitigating the risk of laser-induced coating-material damage and photobleaching. Power-dependent measurements are less susceptible to temperature effects than interferometric techniques, and place loose requirements on the laser source. This review highlights the key developments in this area, while discussing the benefits, challenges as well as future development.

## 1. Introduction

Fiber-optic sensors are supporting the establishment of smart cities and improved biomedical diagnostics. Multimode fibers (MMFs) have been extensively used as both the sensor head and the means to deliver/collect light [1,2], due to their high simplicity, high robustness, and wide availability at a low cost. However, the evanescent field interactions along such fibers are typically relatively weak [3,4] compared to other waveguide structures, such as micro/nanofibers [5], or those employing surface plasmon resonance [6], which results in poorly performing evanescent-wave-based sensors. If improved, such sensors can be applied to the detection of chemicals traces and other high-precision applications. To boost the sensitivity, the larger number of total internal reflections of skew rays and their excellent coverage of the fiber surface have been exploited for a variety of applications involving chemical or physical measurands. Such measurements analyze the optical power rather than the phase, and thus are less susceptible to temperature effects than interferometric techniques [7,8]. Furthermore, this power-based sensing technique has very low requirements on the laser source [9,10]. This review presents a general view as well as a coverage on some of the recent key developments in this area, while also discussing the benefits, challenges and prospects.

## 2. Basic Principles

### 2.1. Definition

**Figure 1:**
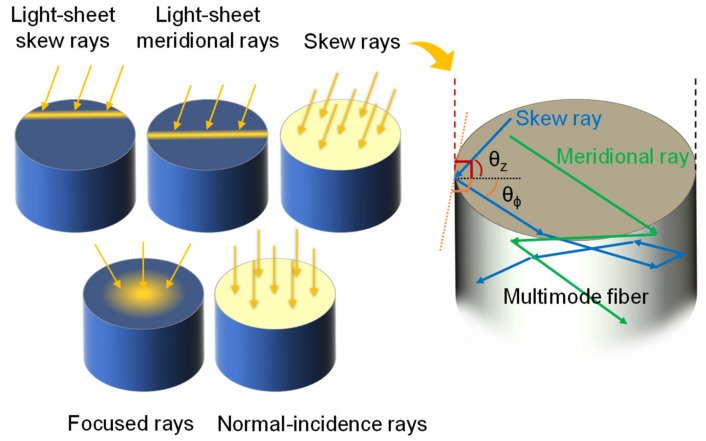
Comparison of different excitation methods. Inset: propagation behavior for different ray types.

**Figure 2:**
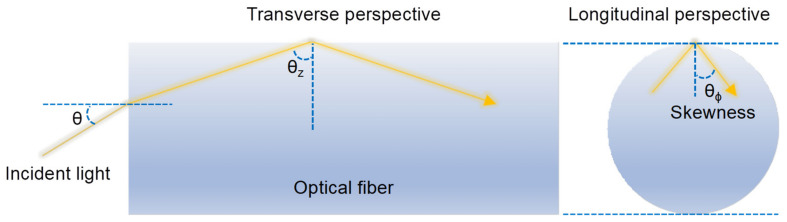
Comparison of different excitation methods. Inset: propagation behavior for different ray types.

The evanescent fields of higher-order modes propagating in an optical fiber are known to extend farther beyond the core–cladding interface (e.g., glass-polymer or glass-analyte) than that of the fundamental mode, which can increase the depth of light–matter interactions on the fiber surface and enhance the sensitivity of evanescent-wave-based sensors [11,12,13]. Skew rays, in some situations, are referred to as leaky rays or tunneling rays, do not intersect the axis of the waveguide. A certain skewness [14] can be imparted on meridional rays when they reflect off a curved waveguide interface, for example, launching light into an optical fiber through one end (i.e., not at the middle segment of its cross-section parallel to the direction of incident light which creates meridional rays). The resulting skew rays can be mathematically described using two angles relative to the waveguide–external interface seen from the longitudinal and transverse perspectives, as shown in Figure 1 and Figure 2.

As long as the refractive indices of the waveguide–external interface facilitate total internal reflections, skew rays can still be guided in the same manner as meridional rays. However, they experience more reflections and thus a higher attenuation. Skew rays enable a much larger number of interface reflections (*N_r_*) than meridional rays under similar conditions, as shown in Equation (1) [15]. This can increase the area of light–matter interactions on the fiber surface, and enhance the sensitivity of sensing mechanisms such as optical-confinement loss and pump/probe light absorption.

(1) $$N_{r}=1/\left(\left[\frac{Dcos\theta_{\phi }}{cos\theta_{z}}+d\right]\times sin\theta_{z}\right)$$

where *D* is the fiber core diameter (i.e., cladding is the functional coating); *d* is the penetration length per reflection (i.e., Goos-Hänchen shift); $\theta_{z}=\frac{\pi }{2}-sin^{-1}\left(\frac{sin\theta }{n}\right)$ is the angle between the ray and the normal of the waveguide–external interface seen from the transverse perspective; $\theta_{\phi }=\frac{\pi }{2}-{cos}^{-1}\left(\frac{i}{N}\right)$ is the angle between the ray and the normal of the waveguide–external interface seen from the longitudinal perspective, which represents skewness (e.g., low skewness: zig-zag path, high skewness: helical path); *n* is the refractive index of the optical fiber; *i* represents the step offset from the fiber center; and *N* denotes the total number of steps from the center to the edge of the optical fiber.

### 2.2. Relation to Modes

In some cases, the near-field intensity profile of light inside a waveguide can be interchangeably described by rays and modes [16]. Hence, a particularly sensitive skew ray group could be recreated using a high-order mode(s) in conventional multimode evanescent-wave sensors [16]. In this sense, skew rays are similar to orbital angular momentum (OAM) modes [17]. In this review, we focus on the ray-optics approach due to its practical advantages of a simple set up, readily tunable for optimization, and a potentially inexpensive build. In comparison, the creation of high-order modes may require complex/expensive phase plates or a computer-controlled spatial light modulator.

### 2.3. Practical Issues

**Figure 3:**
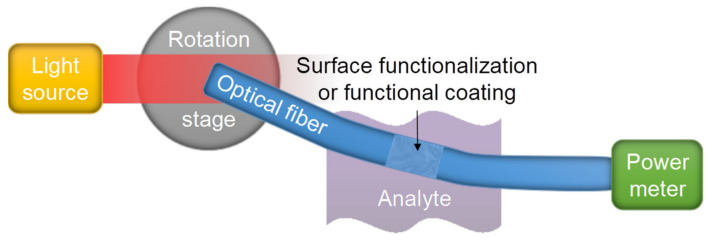
Illustration of the experimental setup of a typical skew-ray-based optical sensor.

Optical all-core/coreless/rod fibers must be highly multimoded and exhibit a lower refractive-index external cladding/environment in order to guide skew rays with a high degree of skewness. In practice, unless the launch conditions are precise and the waveguide geometry is flawless, some rays will become leaky rays due to the lack of total internal reflection. Another fraction of rays may spread into skew rays with different skewness, which can be represented by the concept of mode diffusion. In all cases, the effects of dispersion exist. Inter-modal coupling can be reduced by ensuring a smooth waveguide–external interface, and no deformation of the waveguide geometry. This minimizes the scattering of rays that changes their propagation angles and thus near-field intensity profile. A typical skew ray sensor shown in Figure 3 operates by submerging a section of the sensing fiber in an analyte, with the input end excited by an angled light source, and the output end delivers power-modulated light into a power meter.

### 2.4. Comparison to Other Sensor Designs

In comparison, evanescent-wave-based sensors exploiting coated fiber-tip, optical microfiber/nanofiber or macro-bending enable stronger interactions between light and matter for the same interaction length [18], but risk laser-induced coating damage or photobleaching for achieving adequate signal-to-noise ratios [19]. Conventional multimode evanescent-wave-based sensors facilitate weaker light–matter interactions but mitigate coating damage or photobleaching. Interferometric sensors operate with low optical-powers and offer exceptionally high sensitivity, at the cost of high cross-sensitivity to many other parameters, such as temperature and vibrations [20], not to mention strict requirements on the light source. Optical sensors employing skew rays exploit a uniform spread of light–matter interactions, which exhibit a congregation of qualities (e.g., high sensitivity, high compactness, high optical-stability, high power threshold, handling robustness) with a shortcoming being a lack of extreme sensitivity. Sensing mechanisms based on the transmitted optical power (i.e., minor susceptibility to the thermo-optic effect) are, in this case, considerably more temperature insensitive compared to interferometry based on the phase of light (i.e., major susceptibility to all thermal effects). Lastly, skew-ray sensing techniques place less stringent requirements on the laser/light source, in terms of coherence, linewidth, center wavelength and temporal characteristics.

## 3. Theory

### 3.1. Angle Optimization

This section is a step-by-step discussion of the factors that are considered for generating optimum angles for skew rays used for sensing. As mentioned previously, skew rays undergo more reflections, particularly at higher ray-skewness (i.e., larger $\theta_{\phi }$), compared to that of meridional rays under the same *θ* and fiber length. Moreover, higher-order skew rays can be created at *θ* exceeding (i.e., smaller $\theta_{z}$) that of meridional rays to further increase the number of reflections. This leads to a multitude of advantages: (a) longer total penetration path length inside a functional coating or an external medium, which generally brings about higher sensitivity to external changes [21]; (b) More complete coverage of the fiber surface, which makes it more likely to find local perturbations [22]; (c) better measurement repeatability and improved measurand averaging in the case of non-uniform analytes. However, high-order (small $\theta_{z}$) low-skewness (small $\theta_{\phi }$) rays are known to exhibit larger proportions of power in their evanescent fields due to smaller incident angles, which supports a high sensitivity. Taking the balance into account, high-order medium-skewness rays in general offer a better solution. It is also known that higher-order rays lead to longer optical path lengths and thus accumulate mode dispersion and mode diffusion, which slowly erodes the purity of the optimum ray group. Therefore, by considering the balance once more, medium-order medium-skewness rays (i.e., medium *θ_z_*, medium *θ_ϕ_*) possess the best balance to achieve the highest sensitivity.

It has been proven that, for a certain *θ*, skew rays with different skewness propagate with the same path length. Mathematically, the absence of *θ_ϕ_* in the expression for the total path length per unit fiber length (*L*) confirms this [23]:

(2) $$L=number\;of\;reflections\;per\;unit\;fiber\;length\times path\;length\;between\;reflections\;=\frac{cot\left(\theta_{z}\right)}{Dcos\left(\theta_{\phi }\right)}\times \frac{Dcos\left(\theta_{\phi }\right)}{cos\left(\theta_{z}\right)}=\frac{1}{sin\left(\theta_{z}\right)}$$

### 3.2. Ray Modeling

One of the earliest theoretical papers introducing the concept and modeling of skew rays was reported by Potter et al. [24,25]. In particular, the light collection properties [24] were investigated, resulting in a definition of an effective numerical aperture considering skew rays. An insight into internal surfaces was gained from the experiments: “Since the absorption seems to be essentially the same as in bulk glass, it can be concluded that there is no strong effect in a glass fiber, such as bulk scattering, which is not present in the bulk material. However, the combination of decollimation effects and a reduced reflectivity indicate that there are processes taking place at the internal surfaces of clad fibers which cause them to deviate somewhat from the results of strict geometrical expectations.” In addition, the far-field profile [25] allowed for the derivation of fiber parameters, such as internal reflectivity and the absorption coefficients. Alternative modeling in recent years was presented by Feuermann et al. [26], who quantified the imperfect process of total internal reflections that results in optical leakage even within the numerical aperture of the optical fiber. They concluded: “Total internal reflection at the core-cladding interface in optical fibers is an imperfect process. Even with the best materials, a small fraction of incident light is absorbed in the cladding. Alternatively, the imaginary component of the refractive index may be orders of magnitude smaller than its real component, but is still non-zero. Although the leakage loss at each intersection with the core-cladding interface may be extremely small, the number of reflections can be large, and the net effect is non-negligible leakage”. Regarding applications, they advised: “The problem is especially pronounced for the large NA broad-spectrum multi-mode fibers needed in solar fiber-optic concentrator applications”.

Chen et al. [27] attempted to combine and streamline several models into one for predicting the transmitted power as well as near/far-field profiles. The results achieved good agreement between simulations and experimental data, but a wider variety of optical fibers must be measured to validate the modeling. Further studies on ray modeling were undertaken by Pike et al. [28] and Su et al. [29]. Pike et al., analyzed the propagation of rays inside capillaries and their brightness distribution. The observed ring intensity distribution can provide a quantitative measure of tube bending, though polarization effects (e.g., Brewster’s angle) and the Goos-Hänchen effect was not considered in the modeling. They summarized their work as: “The exact geometrical optics of all rays emitted by any point on the rim are considered, and it is shown that after a single reflection all such rays appear to arise from a single cardioid curve. When rotated about the optic axis, this cardioid predicts the flaring ring intensity distribution observed”. Su et al., found that the near-field profile (NFP) of the output beam is a function of the launch conditions, and established a theoretical model for step-index fiber with an adjustable ratio of meridional modes and skew modes. They have concluded that: “large-core fibers the output NFP is a strong function of beam launching conditions such as input spot size and alignment. When the spot size is substantially less than the core size, and it is aligned accurately to the fiber axis, then meridional modes are excited preferentially. When power is coupled into the fiber away from its axis, either by using a large spot size or by misaligning the spot from the fiber axis, then skew modes are excited preferentially”. Furthermore: “The output NFP depends on the phenomenon of mode coupling and is thus influenced by the presence of bends in the fiber”. Commenting on practical fiber coupling, they stated: “Practical beam delivery systems often employ launch optics designed to deliberately underfill the fiber. That is, they are designed to produce an input spot size less than the fiber core size. Such an approach is undesirable when an accurately controlled output NFP is required, because any misalignment occurring at the input will affect the profile at the output”.

Zubia et al. [30] advanced the theoretical framework to simulate multi-step-index fibers. The comprehensive work covered coupling losses due to different types of mismatch and eccentricity. In addition, the authors have carried out a numerical analysis of the pulse dispersion and bandwidth in certain fibers. They assumed a uniform power distribution across the cross-section of the optical fiber, thus resulting in an overestimate of the attenuation. They have carried out additional modeling, including: “Numerical analysis of the pulse dispersion and bandwidth in multi-step index fibers under different light source configurations, taking into account the influence of tunnelling rays. The obtained results have revealed, on the one hand, variations in fiber bandwidth as a direct consequence of the multilayered refractive index profile inherent to these fibers, and, on the other hand, a higher sensitivity of the fiber bandwidth to the offset of launch position when the source is made smaller”. Allington-Smith et al. [31] carried out studies on the effects at the fiber ends, such as mode diffusion, which include practical considerations. However, these studies are limited to cases where the optical fiber is bonded to the supporting ferrule with adhesive. They reported a new model to: “Predict the thickness of rings produced in the far-field with collimated illumination and explained other puzzling phenomena encountered in fiber testing. These data are commonly used to assess the quality of fibers used in astronomy where limiting performance is critical for upcoming cosmology projects such as Dark Energy Spectroscopic Instrument”.

**Figure 4:**
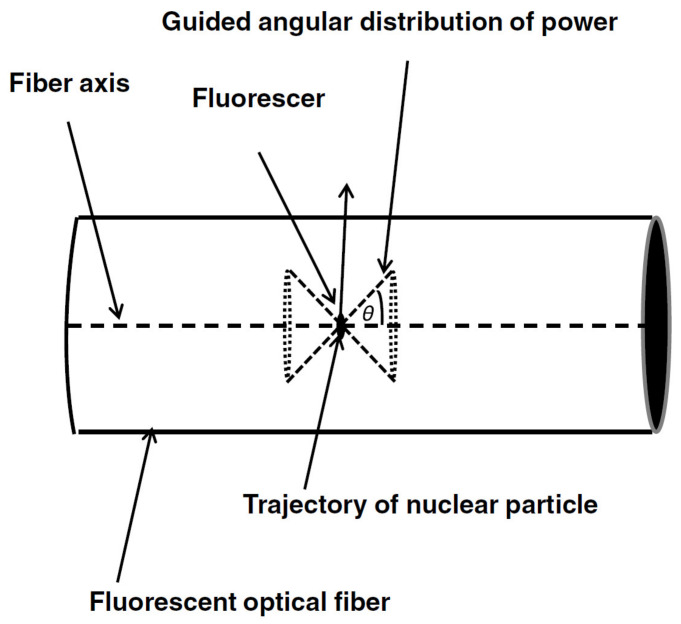
Emission of guided radiation from an on-axis fluorescer within a fluorescent optical fiber. Reproduced with permission [32]. Copyright 2015, SPIE.

In response to the growing field of immunoassay, upconversion and other techniques involving the excitation and measurement of fluorescence, Weiss et al. [32] conducted ray modeling to optimize the trapping efficiency of fluorescence (Figure 4). An expression was formulated to describe the relationship between trapping efficiency and the ratio of the core/cladding refractive indices:

(3) $$TE=\left(P_{W}+P_{N}\right)/4\pi^{2}a^{2}\rho pI$$

(4) $$P_{W}=8\pi^{2}a^{2}\rho pI\left(\frac{n_{cl}}{n_{co}}\right)\left[1-\left(\frac{n_{cl}}{n_{co}}\right)\right]$$

(5) $$P_{N}=P_{W}\left(\frac{n_{co}}{n_{cl}}\right)$$

where *a* is the core radius; ρ is the volume density of fluorescers; *I* is the stimulus (e.g., optical power per unit area or the number of particles crossing a unit area per unit time); *p* is the probability of emission per unit stimulus and per unit solid angle; *n_co_* and *n_cl_* are the refractive indices of the core and cladding respectively. They concluded: “Skew rays that would not be guided if they were meridional rays are not guided in a round fiber, even if they satisfy the condition for total internal reflection at the point of incidence. The reason for this is because for skew rays, the boundary is curved in their plane of incidence and Fresnel’s laws of reflection are valid only for a planar boundary. The result is that although these rays are predicted to experience TIR, they lose energy by tunneling out of the core”. It would be beneficial to see an additional study on the impact of fiber length on the trapping efficiency, as well as potential reabsorption/scattering of the fluorescent signal along the optical fiber.

Chen et al. [33] accompanied basic ray modeling with the characterization of active fibers (e.g., fiber lasers) to determine the optimum excitation angles for specific designs. Although several different active fiber types were tested, it would be more useful to observe the performance impact on fiber lasers. They summarized their findings as: “The quasi-octagonal fiber produced the highest and most consistent pump-absorption at certain launch angles when its corners are aligned with the tilt plane of the pump light, followed by when its flat sides are aligned with the tilt plane. The D-shaped fiber was less effective due to a combination of mode diffusion and core overlap. This technique has potential value to fiber-laser manufacturers, as it can enhance the performance of active fiber components by simply tuning the launch angle of the pump light from a high-brightness laser”. Examples of complete theoretical models are not covered in this review due to the considerable variation in approach (e.g., approximations) for ray optics, unlike those of wave optics modeled using Maxwell’s equations.

### 3.3. Ray Attenuation

Insights into the various optical losses experienced by rays were presented by Snyder et al. [14], Pask et al. [34], Di Vita et al. [35], and Dugas et al. [36]. Snyder et al. derived a concise analytical expression for the loss of all weakly attenuated rays, which could serve as building blocks for transmission systems. One of the main challenges was highlighted: “We cannot, in analogy with bound modes, associate an unbound mode with rays that undergo partial reflection. The fields of an unbound mode are formed by scattering of a cylindrical wave from the circular fiber. An integral of unbound modes is required to represent a partially reflected ray. This is one of the practical inconveniences of the formal analysis. To retain the ray picture, we must approximate the radiation field by leaky modes”. Their solution was: “The attenuation of each ray that forms a leaky mode is the same as the attenuation of the mode. This is because all rays of the family are incident on the waveguide boundaries at the same angle to the normal. The words modes and family of rays are therefore used interchangeably. The attenuation of leaky modes is found here by solving for certain complex roots of the fiber eigenvalue equation”. Di Vita et al., focused on the attenuation mechanisms for skew rays and discussed the causes behind failed excitation of skew rays. Future work could build on this foundation to consider the surface effects of nanoparticle coatings. They deduced that: “Intrinsic mechanism of extinction, even if it influences only leaky rays, is not the only important cause of loss for such rays. In fact, one can see how most of the leaky power loss (~50%) happens in the first few centimeters of the fiber, while the leaky rays which survived the first metres manage to reach lengths of some km, losing only small amounts of residual power. There are many other phenomena which can act on the content of leaky rays in a fiber: some of them (as the presence of an absorbing core, or of a more directive source) can have a small importance. On the contrary some other phenomena can greatly modify the contribution of leaky rays. Among them the presence of a lossy cladding is the one which can act most heavily, especially in step-index fibers. In such fibers the presence of an imperfect core-cladding interface can considerably reduce the amount of leaky power present in the optical waveguide. Among the failed excitation phenomena of leaky rays the ones which give the most noticeable effects (in weakly guiding fibers) are, in order, the presence of a source smaller than core section and the separation between source and fiber; both these phenomena give most noticeable effects in step-index fibers”.

### 3.4. Bend Loss

Kovacevic et al. [37] investigated the impact of bending on the total optical loss, using the explicit finite-difference method to solve the Fokker–Planck power-flow equation. The work focused on bend effects, neglected polarization effects (e.g., Brewster angle) and the contribution from the Goos-Hänchen effect. They showed that: “Power loss induced by bending the fiber to a number of curvature radii. The distribution maxima for bent fibers shift toward the convex side of the bend”. Aslund et al. [38] discussed the angle-dependency of ray losses, and attributed high optical-loss to the diffractive tunneling of skew rays undergoing frustrated internal reflections. The work does not consider the contribution of different ray skewness, which would have largely varying behavior with lower-skewness rays (i.e., larger angles of incidence) experiencing more tunneling than higher-skewness rays. They stressed the importance of considering skew rays: “These rapid losses of skew ray radiation would not be detected in a standard numerical-aperture measurement setup that only considers meridional ray launch. The importance of these skew rays cannot be underestimated: for some applications, such as cladding pumped fiber lasers where a significant part of the light is unavoidably launched skew, these rapid losses of skew light can cause significant interaction of pump light with the external polymer cladding generating heat. This can degrade laser efficiency and limit the total power that can be practically generated for example”.

Ray tracing is undoubtedly a growing and important field for numerous applications in photography, solar concentrators and realistic simulations (e.g., games). Achenbach et al. [39] and Ang et al. [40] carried out extensive simulations with ray racing. Achenbach et al., established the groundwork for a numerical approach (e.g., Monte Carlo) to calculate trapping efficiencies for different types of rays in active fibers, which also applies to bent fibers. They showed that: “Loss of photons due to the curvature of the fiber is a simple function of radius of curvature to fiber radius ratio and is <10% if the ratio is >65. The simulations also show that for larger ratios this loss takes place in a transition region (~π/8) during which a new distribution of photon angles is established. Photons which survive the transition region then propagate without further losses”. Ang et al., rederived the numerical aperture for skew rays using two alternative techniques (i.e., Pauli identity and Euler’s theorem). They showed that: “Light rays inside the optical fiber trace a polygonal helical path characterized by three invariants that relate successive reflections inside the fiber: the ray path distance, the difference in axial distances, and the difference in the azimuthal angles”.

### 3.5. Mode Conversion Coefficients

**Figure 5:**
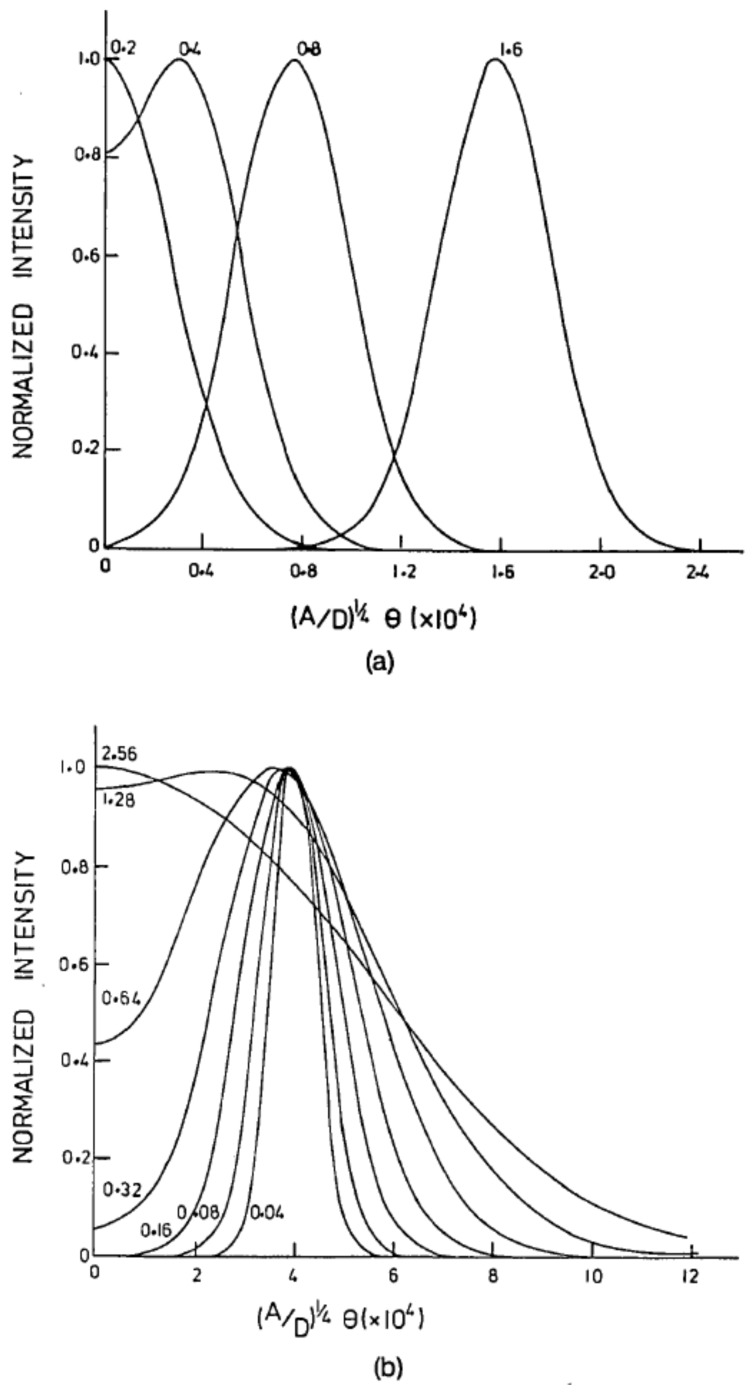
(**a**) Normalized output angular power distribution for normalized input angles. (**b**) Normalized angular power distributions for various fiber lengths. Reproduced with permission [41]. Copyright 1975, The Optical Society.

A variety of other advances in knowledge in this field was made possible by various research groups, including Gambling et al. [41] who studied mode conversion coefficients (Figure 5) from only the far-field profile, and confirmed the validity of Gloge’s power flow equation. Such coefficients are useful for quantifying the level of inhomogeneities or micro-indentations. It was deduced that: “In a liquid-core fiber the normalized mode conversion coefficient is *D* = 3 × 10^−6^ rad ^2^ m^−1^ and increases by as much as an order of magnitude when transverse pressure is applied. In glass-core fibers the mode conversion coefficient is some 2 orders of magnitude larger than in the unstressed liquid-core fiber and in one sample was nearly doubled by heat treatment. The good agreement between theory and experiment confirms the validity of Gloge’s power flow equation and the assumptions on which it is based, in particular that coupling occurs primarily to adjacent modes”. His team expressed the output distribution of optical power as:

(6) $$P\left(x,z\right)=exp\left[-\left(\frac{x_{0}+x}{2}\right)\left(\frac{1+exp\left(-bz\right)}{1-exp\left(-bz\right)}\right)\right]\times \left[\frac{exp\left(-bz/2\right)}{1-exp\left(-bz\right)}\right]I_{0}\left[\frac{{\left(4x_{0}x\right)}^{\frac{1}{2}}exp\left(-bz/2\right)}{1-exp\left(-bz\right)}\right]$$

where *x* and *z* are coordinates along the transverse and longitudinal directions respectively; *b* = 4(*AD*)^1/2^; x_0_ = 4(*AD*)^1/2^*θ*^2^ is the normalized launching angle of incidence; *A* = second-order coefficient in the expansion of the loss coefficient due to absorption and scattering; *D* = *d*_0_(*λ*/4*an*)^2^; *d*_0_ = zero-order term of the coupling coefficient; and *a*, *n* = the core radius and refractive index, respectively.

### 3.6. Impulse Response of Fiber

Cozannet et al. [42] investigated the impulse response contribution of skew rays and concluded that skew rays can be neglected in the case of solid-state laser sources. Barrell et al. [43] presented a study on the optimization of lens excitation of skew rays and discussed the accuracy of ray optics to model light propagation in MMFs. It was found that for higher V numbers (i.e., mode capacity), ray optics become increasingly accurate. However, it is not clear if the imaginary component (i.e., loss component) of the refractive indices were considered. They summarized their findings as: “We have discussed the excitation of an optical fiber by a plane wave focused through a lens. In particular, we have investigated the selection of the optical system parameters with a view to maximizing the power coupled into the fiber. Also included are the effects of various mismatches in the optical system, e.g., transverse, longitudinal and angular misalignment of the lens and fiber axes. The accuracy of various approximate analytical methods, e.g., geometric optics in MMFs and Gaussian modal fields in monomode fibers, has been investigated by comparison with the full electromagnetic modal analysis”.

### 3.7. Angular Momentum

Since skew rays can be made similar to specific OAM modes, Hashimoto et al. [44] analyzed the angular momentum of skew rays and showed that the skew rays polarization states in a graded-index fiber are invariant for high frequencies but not for low frequencies. They showed that: “Certain circularly polarized skew rays are connected to oppositely rotating circularly polarized skew rays as the frequency varies beyond the critical frequency. The states of the polarization are quickly connected to each other in a narrow region of frequency and, in rough approximations, such as in perturbational analyses and in lower-order asymptotic analyses, this phenomenon disappears”. Herskowitz et al. [45] undertook further investigation of skew-ray power flow along an optical fiber. They attributed the anomalous polarization to the inhomogeneity of the medium. They discussed their results: “Measurements of the width of the angular distribution as a function of the fiber length and the launching angle were found to yield very good agreement with the solution to the time-independent power flow equation. The power loss associated with the mode-coupling mechanism of this model was also calculated. A method has been devised to determine the coupling constant of a test fiber. The analysis presented in this paper for step-index fiber may be generalized to any index profile by replacing the axial angle by mode order”.

### 3.8. Caustics

Other notable works were contributed to by authors such as Adler et al. [46] on the visualization of high-order caustics as a result of skew rays. They observed: “Expected interior caustics, but we observe much more as well. Because the internal reflection Fresnel coefficients at diagonal incidence are much larger than those at normal incidence, we are able to see relatively high-order caustics. Because these reflection Fresnel coefficients peak strongly over a narrow range of ray impact parameters near the edge of the cylinder, individual noncaustic ray trajectories become visible as well. Because our glass rod has small imperfections at its surface and weak inhomogeneities of the glass, yet another class of caustics appears”.

### 3.9. Speckle Field

Bolshtyansky et al. [47] considered generation of speckle fields from superimposing skew rays with reference light. They concluded: “We were able to generate a speckle field with finite vorticity, i.e., with unbalanced densities of positive and negative vortices, through transmission of a laser beam through a multimode optical fiber as a group of skew rays. The statistical calculation allowed us to express the vorticity through the curl of the average direction of light in the beam. The theory showed reasonable agreement with the direct measurement of vorticity. The experiment also showed that the particular fiber does not mix +*m* and −*m* modes considerably, at least at the distance used, 1.2 m”.

### 3.10. Vortex Lens

Johnson et al. [48] used a vortex lens to efficiently couple light into skew rays propagating in a graded-index fiber. They summarized their work as: “An analytical model was developed for the amplitude and phase of the corresponding point spread function of the vortex lens system. Results were used to verify the coupling effects as a function of focal length and vortex lens number. Additional computations were performed for a graded index fiber with an on-axis perturbation. The results demonstrated that the vortex launch minimizes the pulse width degradation experienced by on-axis profile distortions”.

## 4. Sensors Overview

### 4.1. Measurands

**Figure 6:**
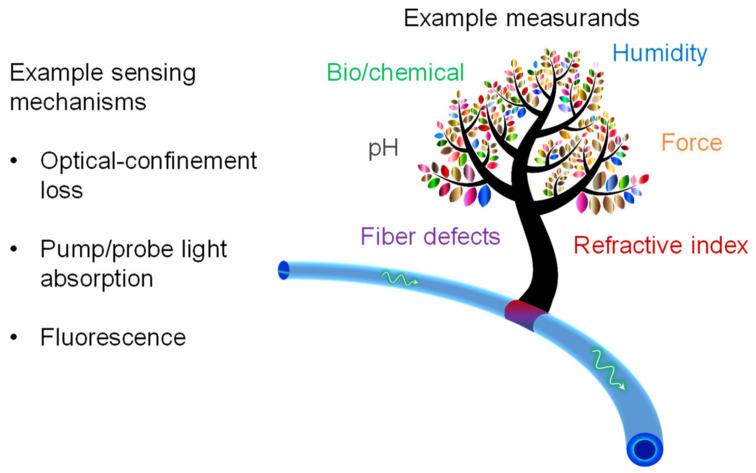
Illustration of expected high-value regions of the detected parameter or sensitivity.

Multimode all-core/coreless/rod fibers are readily available from many vendors and have been used in evanescent-wave-based bio/chemical sensors for delivering light and probing analytes. With a lower-index coating, they can support the propagation of skew rays as well as their evanescent interactions with the measurement environment. Such fibers offer several benefits, including simple structure, considerable ruggedness, and low cost. The resulting sensors are typically all-rounders in terms of sensitivity and practicality. The list of measurands explored in the literature range from chemicals to humidity to fiber defect, as illustrated in Figure 6.

### 4.2. Sensor Optimization

**Figure 7:**
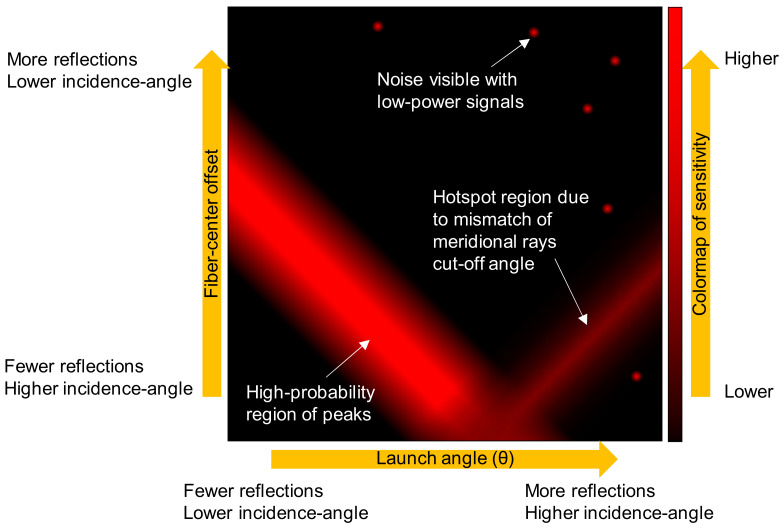
Illustration of the contribution to *θ_ϕ_* from the fiber-center offset and the input light angle.

As a sensor, a relationship between the measurand (e.g., analyte concentration) and the detected parameter (e.g., attenuation) must be established through lab tests to provide calibration data that can be used to provide a readout of the measurand when given the detected parameter. For a systematical approach to optimize the performance, the configuration parameters of *θ* and the fiber-center offset are incremented while the transmitted power is collected for each step of the measurand. Typically, the peak values of the detected parameter are found at high values of *θ*, high values of fiber-center offset, or medium values of both. Figure 7 illustrates that artefact peaks can occur more outward than the actual peaks caused by enhancements in light–matter interactions. These are caused by changes in the cut-off (i.e., loss of optical confinement) of meridional and/or low-skewness rays, due to refractive index changes in the local environment. Such effects are abrupt and appear as a step change rather than a gradual change, which translates into a sudden surge in the detected parameter between a narrow range of the measurand. The calibration graph for the end-user is based on the relationship associated with the optimum launch conditions.

For the purpose of optimization, the parameters of importance are: the wavelength of the input light, fiber diameter, coating thickness/concentration, fiber-center offset (governs *θ_ϕ_*), launch angle (governs *θ_z_*), fiber length, and polarization azimuth of the input light. For chemical sensors, the wavelength of the laser source used for probing must align with an absorption wavelength band of the analyte. For physical sensors, the choice of wavelength is less critical, and often low cost and commercial availability plays a significant part in the decision.

If the detected parameter is transmitted power, the detection limit/accuracy is inversely proportional to the signal-to-noise ratio. For sensing mechanisms not based on interferometry, shot noise (i.e., proportional to the square root of the optical power) is most often the main noise source. Increasing the fiber diameter increases the input optical power by the area, and thus scales the power-equivalent noise. The combined result means the signal-to-noise ratio increases proportionally with increasing fiber diameter. However, increasing the fiber diameter decreases the number of reflections according to Equation (1), which lowers the sensitivity and offsets the first enhancement in detection limit/accuracy. A significant advantage to larger fiber diameter is that it enables a more precise selection of ray groups when a light sheet is scanned across the input fiber end-face, which can optimize the sensitivity. To conclude, a large fiber diameter is generally desirable, and its upper limit depends on the requirement on the application’s requirements on physical flexibility and space occupation.

A thin-walled capillary can also be used to achieve orders of magnitude of enhancement in sensitivity [49]. Skew rays guided in such fashion are identical to those propagating in a solid-core fiber. If meridional rays are used, the thin walls can enable a very large number of reflections. The main advantage of using a capillary is the ability to pull and retain analytes in the hollow region using capillary force or vacuum suction. The drawback is that such hollow structures can break easily and are more susceptible to vibrations and acoustic noise.

Depending on the measurand, the functional coating material is chosen for high sensitivity and/or high specificity. The coating concentration should also minimize self-quenching and photobleaching to extend the lifespan of the sensor head. Any changes in the coating material’s refractive index should be considered for a possible loss of optical confinement.

Since the strongest light-matter interaction (e.g., total penetration path length, the portion of power in their evanescent fields) can be realized with a specific skewness of rays in addition to a certain *θ*, the conventional method of launching a collimated beam and exciting mixed ray groups is not ideal. Instead, a collimated light sheet (i.e., horizontal plane of light) can offer much better precision at pooling resources to the key contributor to the sensitivity. Furthermore, a limited number of ray groups should result in a better stability due to a lack of transverse mode competition [50].

**Figure 8:**
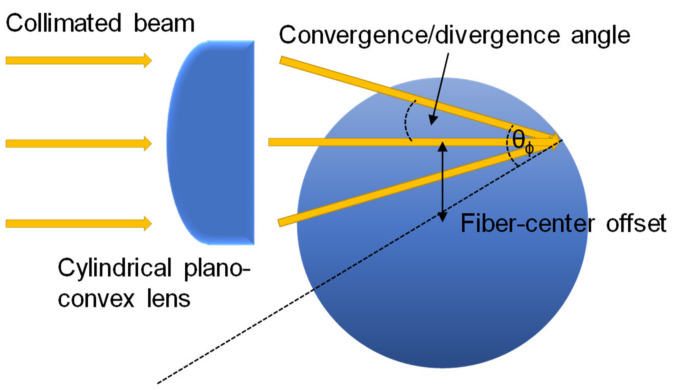
Example variations of the sensing mechanism and measurands for skew-ray-based sensing.

With a light sheet, the angles of *θ_ϕ_* (i.e., fiber center-offset) and *θ_z_* (i.e., via *θ*) can be optimized to deliver the most effective incidence angle and number of reflections in order to boost evanescent-field interactions and thus increase the sensitivity. The fiber-center offset or *θ_ϕ_* can be incremented by vertically translating the horizontal light-sheet incident across the input fiber end-face. As shown in Figure 8, the fiber-center offset and the vertical angle of the input light govern *θ_ϕ_* via the curved interface of the fiber, with the range of *θ_ϕ_* governing the initial thickness of the guided ring of light. For precise excitation of skew rays, the narrowest light ring can be achieved by minimizing the thickness of the input light-sheet, reducing the convergence/divergence angle of the input light-sheet, and shifting the focal point just inside the input fiber end-face. Since some parameters are trade-offs, a good balance is needed. Such fine tuning can be achieved by reshaping a collimated beam using a cylindrical plano-convex lens into a light sheet.

Although the free-space divergence of light is not a concern inside an optical waveguide due to the transition from Gaussian beam propagation to circular-track propagation inside, other effects preside. Mode dispersion (i.e., geometrical, chromatic dispersion) and mode diffusion (i.e., Rayleigh scattering, Mie scattering) accumulate with increasing optical path length of light along the sensing fiber. This gradually transforms light-sheet skew rays from a thin-line input to a thick-ring output. While the ring-like near-field intensity distribution is beneficial, as it improves coverage of the fiber surface, the thickness of the lightsheet should be kept minimal. A thick light ring lowers the ray-group purity by the conversion to non-optimum ray groups.

The polarization azimuth of linearly polarized input light can be rotated to maximize sensitivity via a good balance between penetration depth and transmission. Lastly, aligning the center of the sensing fiber and the beam (i.e., approximately rectangular overlap with fiber) offers better mechanical stability and rotation-center tolerance.

### 4.3. Refractometers

**Figure 9:**
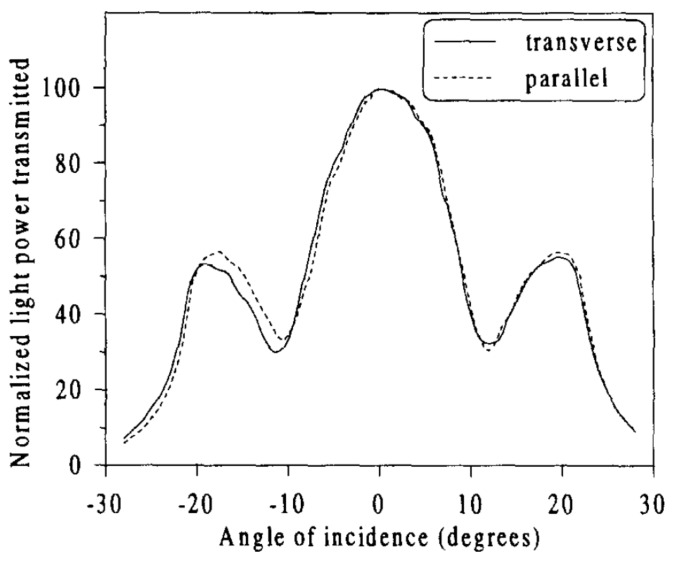
Experimental curves of the normalized angle-resolved power for different polarization states [51]. Copyright 1996, Elsevier.

The first sensors exploiting skew rays for sensing were refractometers, shown by Archenault et al. [21] and Ronot-Trioli et al. [51], who reported detection limits of ~10^−4^ (1.405–1.457 RIU) and 3.5 × 10^−5^ RIU (1.355–1.410 RIU), respectively. Archenault et al., explored the acceptance angle of a MMF for measuring refractive index. They discovered that: “The intensity of the light transmitted through a MMF illuminated with a collimated beam is very sensitive to small variations of the refractive index of a liquid acting as the cladding of the fiber”. Ronot-Trioli et al., utilized surface plasmon resonance (SPR) to enhance the detection limit, and optimized the coating thickness. They observed ripples in the angle-resolved power spectral (Figure 9), induced by surface plasmons. They concluded: “Skew rays propagated in the fiber can undergo reflections at the metal/dielectric interface with the surface plasmon resonance coupling angle. At this specific angle, the transmitted light power is attenuated as a result of the incident optical energy transferred to the SPR charge density waves”. As for applications, they stated: “The use of an optical fiber sensor is interesting for continuous and remote monitoring in industrial processes”.

Lin et al. [52] also adopted SPR with skew rays to demonstrate the quantification of refractive index, spanning 1.33–1.36 RIU. Since a monolayer of long-chain thiol was used and optimized to protect the surface of silver from deterioration, the surface is generally more susceptible to damage. Further advances were made by Dwivedi et al. [53] on modeling SPR and skew rays with a focus on signal-to-noise ratios, who reported a decrease in both sensitivity and signal-to-noise ratio with increasing skewness. They attributed it to: “The sensitivity is better in the case of gold, whereas silver demonstrates better SNR. This occurs because gold has larger values of real as well as imaginary parts of dielectric constant in comparison with silver at any wavelength”. Singh et al. [54] used a light-emitting diode to excite skew rays for SPR (1.33–1.36 RIU), and simulated the designs based on core diameter, length of the sensing region, and numerical aperture of the fiber. It was concluded that a collimated or focused light source offered better sensitivity, but the diffuse light source enabled higher compactness and lower cost. Taking it a stage further, a light-diffusing fiber was found by Cennamo et al. [55] to be suitable for exciting SPR modes with random ray groups, who reported a sensitivity of 4000 nm/RIU. Interestingly, the diffusion effect leads to a higher sensitivity than ordinary-fiber SPR sensors. The authors believe it to be caused by excitation of a higher number of modes, and a more extensive analysis is needed to explain the enhancement. The expression for the normalized transmitted power is:

(7) $$P_{L}=\frac{1}{2}\left(\frac{\sum_{\theta_{i}=\theta_{c}}^{\pi /2}P_{0}\left(\theta_{i}\right)\times R_{p}{\left(\theta_{i}\right)}^{N\left(\theta_{i}\right)}}{\sum_{\theta_{i}=\theta_{c}}^{\pi /2}P_{0}\left(\theta_{i}\right)}+\frac{\sum_{\theta_{i}=\theta_{c}}^{\pi /2}P_{0}\left(\theta_{i}\right)\times R_{s}{\left(\theta_{i}\right)}^{N\left(\theta_{i}\right)}}{\sum_{\theta_{i}=\theta_{c}}^{\pi /2}P_{0}\left(\theta_{i}\right)}\right)$$

where *P_0_* (*θ_i_*) is the initial power propagated by total internal reflections; *N(θi)* = *cot(θi)L/d* is the number of reflections, *d* is the core diameter; and *R_p_* and *R_s_* are the Fresnel reflectance coefficients at the boundary between the core and the sensing medium for p-polarization and s-polarization, respectively.

Chen et al. [56] explored new fiber designs including the use of a GTwave fiber to facilitate single-ended measurements, realizing a detection limit of 4.9 × 10^−5^ RIU (1.33–1.43 RIU). Such specialty fibers with two coupled rods inside the same cladding are typically used for mixing signal and pump light for the generation of high-power lasers. A long length of optical fiber prior to the sensor head is needed to ensure stable coupling between the two cores. In addition, the exposed section may need adhesives to fix the cores together in turbulent environments in order to prevent changes in the coupling. This design can be further exploited with the assistance of metal surface functionalization to increase the intensity of the evanescent field in close proximity to the contact regions.

### 4.4. Alkanes

Other types of measurands include alkanes gas sensing by Abdelghani et al. [57], who employed a porous silica coating via the sol-gel method with on uncladded MMF. The material was claimed to possess numerous benefits: “This material presents some advantages compared to more classical polymers. For example, it will be tougher, more inert, intrinsically bound to the core and chemically more resistant”. They demonstrated a detection limit of 0.6% analyte concentration. It was concluded: “The sensitivity and the detection limit for trichloroethylene are lower than for the other chlorinated hydrocarbons and that the sensitivity and the detection limit for chlorinated hydrocarbons are lower than for alkanes”.

### 4.5. Antigen

Lin et al. [58] non-specifically measured the IgG antigen with a skew ray excitation of SPR on multimode optical fiber, which they reported is: “Capable of investigating the averaged optical parameters over entire adsorbed layers”. They reported a detection limit of 70 ng/mL (70 ppb) between a range of 0–1.2 μg/mL. The advantages of using skew rays were stated: “This whole surface probe is very suitable to investigate the kinetics of a surface adsorption or desorption especially their adjustment process, which is rather delicate and hard to be monitored in real time”.

### 4.6. Relative Humidity

The measurement of relative humidity was presented by Khijwania et al. [59], who exploited a U-shaped probe with a CoCl_2_ embedded polyvinyl alcohol polymer coating and demonstrated a response time of 1 s within the range of 1.6–92%RH. It was observed that smaller bend radii and smaller fiber diameters improved the sensitivity, which fortunately are complementary factors. Chen et al. [60] followed up with a new approach of exciting skew rays with a light sheet for systematically finding and adopting the most sensitive ray group to enhance light–matter interactions and thus improve the sensitivity. With a humidity-sensitive polyelectrolyte multilayer coating, the detection limit was shown to be 0.007%RH within a range of 10–94%RH. More material exploration is required to improve the linearity of the optical response to relative humidity.

### 4.7. Aerosol

Steinberg et al. [61] conducted a beam analysis with the help of ray tracing, and comparison for aerosol sensing in biomedical applications. The authors established that ring-like beams are significantly better for capillaries. They summarized their findings: “The Bessel beam shape was proved less adequate for sensing purposes due to its relatively merdional propagation and low SNR compared to a Gaussian and ring-like beams. Ring-like beam proved most beneficial for sensing aerosol particle inside a hollow core waveguide, combining both high degree of skewness and a relatively low power loss”.

### 4.8. pH

**Figure 10:**
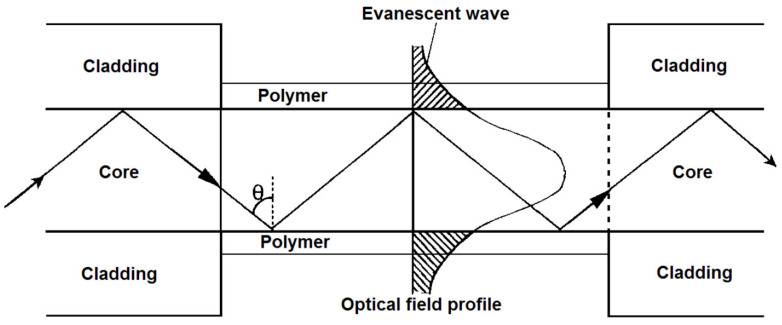
Sensor principle and evanescent wave field (fundamental mode) in an optical fiber. Reproduced with permission [62]. Copyright 2014, SPIE.

Hammarling et al. [62] demonstrated an optical fiber coated with poly (β-amino ester) for measuring pH (Figure 10) in blood. The most significant signal change is between 6 and 7 pH, which is useful as most commercial pH sensors are the least sensitive in this region. The penetration depth of the evanescent wave (amplitude of the evanescent wave decrease to 1/e of its initial value) was expressed as:

(8) $$d_{p}=\frac{\lambda }{2\pi_{1}n_{1}\sqrt{cos^{2}\theta_{z}-{\left(\frac{n_{2}}{n_{1}}\right)}^{2}}}$$

where *n*_1_ and *n*_2_ are the core and cladding refractive index, respectively; and *θ_z_* is the incident ray angle.

### 4.9. Rhodamine

**Figure 11:**
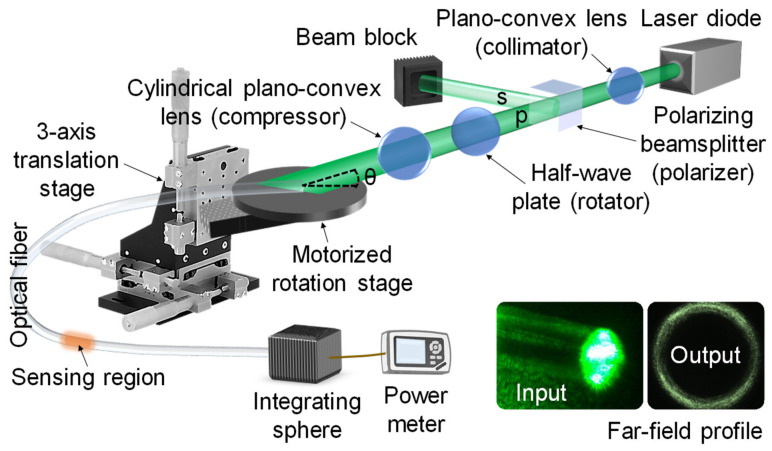
Experimental setup for the enhanced detection and quantification of Rhodamine B. Reproduced with permission [63]. Copyright Year, Publisher.

Chen et al. [63] used a combination of light sheet and skew rays for chemical sensing, as shown in Figure 11. A demonstration with Rhodamine B was conducted, attaining a detection limit of 24.9 ng/mL (24.9 ppb) between 0–1 mg/mL. It is possible to extend the technique to measure other chemicals, or use a functional coating that is specific to certain chemicals. They found that there is: “Consistent attenuation from P-polarized to S-polarized light, which varies by no more than 0.3 dB”. A comparison was made in Table 1 between different excitation methods [63], with light-sheet skew rays showing an enhancement in attenuation of up to one order of magnitude relative to skew rays and at least three orders of magnitude over nearly normal-incidence rays. It is evident that light-sheet skew rays offer the highest sensitivity under the tested conditions.

**Figure 12:**
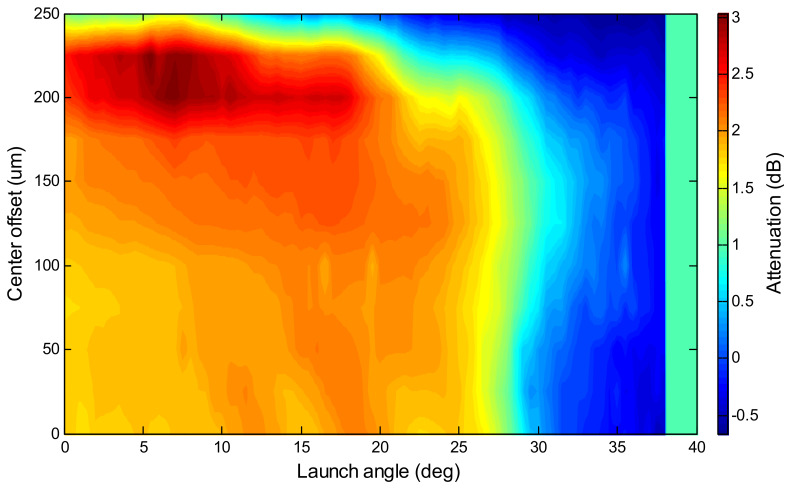
Attenuation (color depth) induced by 0.1 ng/mL concentration Rhodamine B relative to pure water as a function of launch angle and center offset. Reproduced with permission [64]. Copyright 2019, Elsevier.

Chen et al. [64] improved upon the base design by introducing localized SPR to light-sheet skew rays, resulting in an enhancement in attenuation (3.08 dB with 0.1 ng/mL and 3 mm length, or 1.18 × 10^8^ dB/(mg/mL)/cm) of up to seven orders of magnitude for 0.1 ng/mL of Rhodamine B under similar conditions. The attenuation peaks are visible in Figure 12. The detection limit can be as low as 0.18 ng/mL (180 ppt) between a range of 0.1–10 ng/mL. It would be possible to increase the sensitivity and improve the detection limit with a liquid dye or functional coating material that does not photobleach. A functional coating may also help to reduce the saturation of gold nanoparticle interactions by limiting the migration of molecules at low concentrations.

### 4.10. Light Source and Coupling

A light source and coupling scheme comparison were carried out by Klein et al. [65], which evaluated low-cost approaches for practical use. They summarized their finding as: “As expected, the coupling efficiency of the broadband light-sources with the excitation fiber needed for higher-order skew mode excitation in the requested spectral region is extremely low”.

### 4.11. Fiber Defect

Although most skew-ray-based sensors involve evanescent field interactions, the same technique can also be extended to measuring physical measurands. Chen et al. [22] demonstrated an inline approach to detect microscopic defects in optical fibers with a lower refractive-index coating for fiber-drawing and fiber-packaging applications. They stated that: “Skew rays facilitate a circular coverage shown along the cladding-coating interface. The combination of these two factors makes skew rays significantly more sensitive to surface imperfections than meridional rays”. Through advanced signal processing [66], the upper radial position can be estimated to indicate whether the defect lies in the core, cladding or cladding-coating interface. This new technique allows for rapid characterization of buried optical fibers, which limit other methods from being practical. Further investigation would be ideal on the impact of stress fields and their relative magnitude compared to physical defects. The outermost position of defects relative to the fiber center can be derived from the doughnut ratio of the near-field intensity profile of light:

(9) $$R_{def}=R_{clad}\left[cos^{-1}\left(\frac{n_{1}}{sin\theta }\times cos\left[sin^{-1}\left(\frac{n_{2}}{n_{1}}\right)\right]\right)\right]$$

where *R_clad_* is the radius of the cladding, *θ* is the launch angle of the incident light; and *n*_1_ and *n*_2_ are the refractive indices of the core and cladding, respectively.

### 4.12. Weight

Skew rays were also applied to force sensing with the help of flexible plastic optical fibers, as shown by Chen et al. [15]. A detection limit of 875 mN within a range of 0–24.2 N was demonstrated. Although just a proof-of-concept, it is clear that the fiber materials of lower Young’s modulus can substantially increase the sensitivity and dynamic range, because they can deform easier and up to a higher limit.

## 5. Discussions

### 5.1. Key Results

With skew-ray-based sensors being a relatively small but emerging field, most findings can be considered important advances that advance the field towards high-performing and practical optical sensors. To list a compact selection of the state-of-the-art results in the field of skew-ray-base sensors: (a) Ronot-Trioli et al. [51], who combined SPR with skew rays to measure refractive indices changes as small as 3.5 × 10^−5^ RIU (1.355–1.410 RIU). (b) Abdelghani et al. [57] introduced skew rays to sensing alkanes, achieving a detection limit of 0.6% analyte concentration. (c) Likewise, Lin et al. [58] exploited skew rays for IgG antigen detection for the first time. They reported a detection limit of 70 ng/mL (70 ppb) between a range of 0–1.2 μg/mL. (d) Chen et al. [61] combined skew rays with a polyelectrolyte coating to increase the responsiveness to RH. They claimed a detection limit of 0.007%RH (10–94%RH). (e) Steinberg et al. [61] conducted a beam analysis with ray tracing to optimize skew rays for aerosol sensing. (f) Hammarling et al. [62] demonstrated the importance of skew rays for pH sensing. (g) Chen et al. [63] compared various fiber excitation methods and showed an improvement in analyte absorption (e.g., Rhodamine B) with light-sheet skew rays (i.e., finer selection) of up to one order of magnitude relative to skew rays and at least three orders of magnitude over nearly normal-incidence rays. Chen et al. [64] demonstrated the advantages of light-sheet skew rays further with measurements on the concentration of Rhodamine B, achieving a detection limit of 0.18 ng/mL (180 ppt) within a range of 0.1–10 ng/mL. (h) Chen et al. [22] introduced the ability of skew rays to detect microscopic defects in fiber core, cladding or coating. (i) Chen et al. [15] implemented a simple weight sensor using plastic optical fibers and skew rays, demonstrating two designs with two different enhancement types (e.g., increase sensitivity or increase angle tolerance of incident light).

### 5.2. Challenges

There are numerous collections of theoretical analysis and modeling for skew rays and their behavior in optical fibers. A unified model is desired to integrate knowledge at the fundamental level up to the system level, in order to make the ray optics approach more accessible and support experimental designs with a comprehensive and consistent groundwork.

As mentioned previously, optical sensors powered by skew rays possess numerous qualities such as high sensitivity, good stability, high power threshold, and are generally robust. Although they lack extreme sensitivity on the level of interferometric sensors, their practical benefits make up for it in some cases. However, for applications requiring the detection of nanoscale perturbations or elongation, interferometers, micro-resonators [67] and surface plasmon resonance [6] among other precision methods are irreplaceable. Other ray-optics approaches to increasing the sensitivity include the possibility of using thick-walled capillaries and meridional rays such that the number of total internal reflections are massively increased along the circumference of the fiber. Alternatively, thin-walled capillaries can offer evanescent field enhancements [49] similar to tapered fibers. However, the mechanical stability of thin-walled structures remains an issue.

Owing to highly divergent skew rays at the output end of an optical fiber, the simplest approach is to implement a double-ended configuration (i.e., transmission), where the total power is capture by an integrating sphere or a large-area photodiode. To achieve a single-ended configuration is more complex, as it requires a beamsplitter at the input end to allow light in and out. A possible solution is to employ a carefully tailored vortex lens [48] to convert the returning divergent rays into a collimated beam for collection. This approach is feasible for an optimized design, but not suitable for the optimization stage where the skewness of rays is frequently changed.

### 5.3. Benefits

Skew rays already amplify the sensitivity many cases, through a larger number of Fresnel reflections. Enhancement stacking is a well-known strategy to combine all possible techniques for maximizing the sensitivity or minimizing the detection limit. For skew rays, combining with functional materials and plasmonics are possible options (e.g., SPR or localized SPR) when pump/probe light absorption is involved. There is an incompatibility with U-shaped fiber bending when the light-sheet excitation of skew rays is used, due to significant mode dispersion associated with changing geometry and possible mode diffusion caused by surface irregularities. For skew rays generated from a collimated beam, where there is already a mixture of ray groups, bending is not an issue. Fiber tapering to increase optical leakage is also not a complementary effect, due to the shrinking upper limit of mode capacity, as well as changing geometry.

The robust nature of thick MMF (>200 μm diameter fiber) lends itself to a high ease of use, as the sensor head is not fragile and can be cleaned more easily than micro/nanofibers. As a result, such sensing fibers can be reused without issues of significant degradation in sensitivity and structural integrity. The drawback of using thick MMF is the lower physical-flexibility and larger footprint that may pose problems in confined spaces.

Most skew-ray-based sensors are potentially inexpensive to manufacture. This is because power-based detection gives rise to very low requirements of the light source (e.g., Lambertian source such as a light-emitting diode) and the photodetector (e.g., photodiode), where parameters related to the center wavelength, coherence and polarization are often very flexible. As a result, there is a potential to create integrated-optics versions of skew-ray-based sensors, operated by low-cost components for incorporation into circuit boards for a wide range of applications. Furthermore, it is possible to embed flexible optical fibers hosting skew rays into smart textiles for personal health monitoring.

### 5.4. Future Development

One can expect further innovations in the area of skew rays with the rapid emergence of new types of specialty fibers (e.g., microstructured, organic) to enhance sensitivity. New optical structures could lend themselves to optical switching or multiplexing with skew rays, by tuning the skewness of rays to control the angle and position of output rays. Another opportunity might be in distributed fiber-optic sensors to improve spatial resolution. One can envisage it as wrapping a linear axis into a coil. The challenge is to filter and regulate the diverse backscattered angles of light to deduce the correct position of interest.

## 6. Conclusions

This review covered the key developments in the area of skew rays, both modeling and sensors, while discussing the benefits, challenges and prospects. Optical all-core/coreless/rod fibers supporting skew rays must be highly multimoded, and feature a lower refractive-index external cladding/environment. In general, optical sensors employing skew rays give rise to high sensitivity, good stability, high power threshold, and reasonable robustness. However, they lack the extreme sensitivity typical of interferometry and are thus not suited for applications involving weak signals. Although decades old, this area still offers much room for innovation (e.g., effect stacking, light propagation in new optical fibers) and new applications (e.g., biomedical).

## Figures and Tables

**Table 1:** Comparison of different excitation types. Repeatability is the percentage error. Reproduced with permission [63]. Copyright Year, Publisher.

Excitation Type	Measured Attenuation (dB)	Repeatability (%)
Light-sheet skew rays (collimated)	34.9 (20.0°, 12.5 µm)	~5
	32.0 (17.0°, 112.5 µm)	
Meridional rays (collimated)	30.2 (21.5°, 0 µm)	~5
Skew rays (collimated)	21.2 (20.0°)	~13
	17.2 (15.5°)	
Focused rays (tilted, centered)	~19 higher than normal-incidence (1.7°)	N/A
Focused rays (centered)	8.5 (1.7°)	~3
Normal-incidence rays	0.9 (0°)	~13
